# Sleeping Beauty transposon system harboring HRAS, c-Myc and shp53 induces sarcomatoid carcinomas in mouse skin

**DOI:** 10.3892/or.2013.2264

**Published:** 2013-01-31

**Authors:** SUNYOUNG JUNG, SIMON WEONSANG RO, GEUNYOUNG JUNG, HYE-LIM JU, EUN-SIL YU, WOO-CHAN SON

**Affiliations:** 1 Asan Institute for Life Sciences, University of Ulsan College of Medicine, Asan Medical Center; 2 Department of Pathology, University of Ulsan College of Medicine, Asan Medical Center; 3 Liver Cirrhosis Clinical Research Center, Yonsei University, College of Medicine, Seoul, Republic of Korea

**Keywords:** phenotyping, mice, Sleeping Beauty, oncogene, tumor, immunohistochemistry

## Abstract

The Sleeping Beauty transposon system is used as a tool for insertional mutagenesis and oncogenesis. However, little is known about the exact histological phenotype of the tumors induced. Thus, we used immunohistochemical markers to enable histological identification of the type of tumor induced by subcutaneous injection of the HRAS, c-Myc and shp53 oncogenes in female C57BL/6 mice. The tumor was removed when it reached 100 mm^3^ in volume. Subsequently, we used 13 immunohistochemical markers to histologically identify the tumor type. The results suggested that the morphology of the tumor was similar to that of sarcomatoid carcinoma.

## Introduction

The Sleeping Beauty (SB) system is a genetically engineered insertional mutagenesis system that consists of two components: a transposon, which is a series of DNA mobile elements flanked by indirect repeat sequences, and SB transposase, which catalyzes the mobilization and reintegration of transposon into mouse genomic DNA (1,2). Integration into the host chromosome provides prolonged expression of the transgene. In addition, the SB system has the advantages of both viral and non-viral vector systems (3). For this reason, the SB system has attracted much attention as a promising delivery system, as well as a discovery tool for cancer-associated genes. Its ability to achieve long-term *in vivo* expression suggests that the somatic integration of oncogenes using SB is a feasible approach to the development of molecularly defined tumorigenic mouse models (4). Although xenograft models and genetically engineered mice are able to mimic human cancer progression (5), the present mouse model systems do not correspond to humans with regard to genetic backgrounds (i.e., different genetic variants, genetic mutations and subsequent protein expression) (6–8). A hallmark of human cancer is genetic complexity, meaning a number of different mutations are commonly involved (9). The complex genetic alterations in different types of cancer cause various histological subtypes and can explain the heterogeneous nature of a given neoplasm (9). Carlson *et al*(4) succeeded in eliciting tumor formation in mouse hepatocytes by hydrodynamic injection of a transposon containing an activated NRAS oncogene. Although those investigators studied the simple feature of the tumors induced by insertional mutagenesis, the exact origins and tumor subtypes have yet to be determined.

In the present study, the c-Myc, HRAS and shp53 oncogenes were delivered with SB into C57BL/6 mice. A malignant undifferentiated tumor was subsequently identified at the injection site on the subcutis of the right side of the lower ventral abdominal area. The nature of the tumor was identified by histological and immunohistochemical methods (Table I).

## Materials and methods

### Animals

Female 5-week-old C57BL/6 mice were purchased from Orient Bio (Yongin, Korea). The mice were housed at the laboratory animal facility at Asan Institute for Life Sciences under specific pathogen-free conditions and used according to the guidelines of the Institutional Animal Care and Use Committee of Asan Institute for Life Sciences.

### Plasmid construction

Plasmids encoding the SB transposase (pPGK/SB13) and transposon vectors (PT2/BH) with multiple cloning sites between two indirect repeat sequences (IR/DRs) were used for this study. Plasmids pPGK/SB13 and PT2/BH were kind gifts from Drs David Largaespada and Perry Hackett at the University of Minnesota. The cDNA encoding either c-Myc or HRAS was inserted into the pCXEGFP plasmid (kindly provided by Dr Masaru Okabe at the Osaka University, Japan) and the transcriptional cassettes were cloned into PT2/BH. PT2/shp53/GFP4, transposon plasmids encoding a short hairpin RNA against tumor suppressor P53, was a generous gift from Dr John Ohlfest at the University of Minnesota. DNA used for injection was prepared using an EndoFree Plasmid Maxi kit (cat. no. 12362, Qiagen) according to the manufacturer’s instructions.

### DNA plasmid injections

Animals received a mixture of three types of transposon and the plasmid encoding the transposase, as detailed above. The molar ratio of transposase-encoding plasmids to transposon plasmids was 1:2. First, three types of transposon were mixed in equal amounts in total 50 μg and then the transposase encoding plasmids was added to the transposon mixture with 50 μl of phosphate-buffered saline (PBS). The DNA mixture was collected with an insulin syringe (31 G) and injected subcutaneously near the right inferior mammary gland. Transposon genes and transposase were regarded to be 7,000 and 5,000 kb in size, respectively, rounded to the nearest kb.

### Animal PET imaging

#### Radiopharmaceutical preparation

Decay-corrected radiochemical yields ranged from 60 to 70% and after high-performance liquid chromatography (HPLC) purification, the radiochemical purity was 98±1.2% (mean ± SD). The specific activity of the [^18^F]Flu-deoxy-glucose (FDG) obtained was >100 TBq/mmol. PET scans were performed using a microPET Focus 120 system (microPET, Concorde Microsystem, Inc.) with resolutions of 1.18 (radial), 1.13 (tangential) and 1.44 mm (axial) at the center of the field of view. Each mouse was injected with 7.4 (0.2 mCi) and 37 MBq (1 mCi) [^18^F]FDG into the tail vein and 10-min static PET scans were obtained. Each mouse was maintained under isoflurane anesthesia during the uptake and scanning periods. A heating pad and heat lamp were used to maintain body temperature at ~37°C. PET images were reconstructed by OSEM2D with a cut-off frequency of 0.5 cycles per pixel. No attenuation correction was applied.

#### Tumor monitoring and necropsy

Mice were carefully examined three times each week to detect tumors. Tumors were measured using a digital caliper. The tumor volume (v) in mm^3^ was calculated using the formula v = L × W^2^/2, where L is the longest diameter and W is the tumor length that is perpendicular to L. When the tumor reached 100 mm^3^ in volume, the mice were humanely euthanized and subjected to a necropsy in which the tumor was excised with the circumferential tissue for histopathological examination.

#### Histology and immunohistochemistry

After macroscopic examination, the excised tissues were fixed in 10% neutral-buffered formalin. The specimens were embedded in paraffin and 3-μm sections were cut and stained using a hematoxylin and eosin (H&E) stain. In addition, immunohistochemical markers [all from Abcam, (Cambridge, UK)] were used to subtype the tumor as follows: CD45 (1:2,000), CD163 (1:2,000) and CD68 (1:4,000) for histiocytic sarcoma; desmin (1:1,000) and myogenin (1:100) for pleomorphic rhabdomyosarcoma; HMB45 (1:1,000) and S100 (1:400) for malignant melanoma, α-smooth muscle actin (α-SMA; 1:400) for leiomyosarcoma; pan-cytokeratin (1:3,000), cytokeratin 7 (1:4,000) and cytokeratin 20 (1:2,000) for undifferentiated carcinoma; and murine double minute 2 (MDM2; 1:1,000) and cyclin-dependent kinase 4 (CDK4; 1:1,000) for pleomorphic liposarcoma (Table I).

#### Detection of the expression of genes

Expression of the c-Myc and HRAS genes was identified by immunohistochemistry with anti-c-Myc (1:1,000, Abcam) and HRAS (1:1,000, Abcam) antibodies, respectively. For the detection of the shp53 gene, an anti-GFP antibody (1:4,000, Abcam) was used as a GFP gene was contained in the shp53 DNA plasmid as a reporter gene.

## Results

### Tumor observation

**Figure 2:**
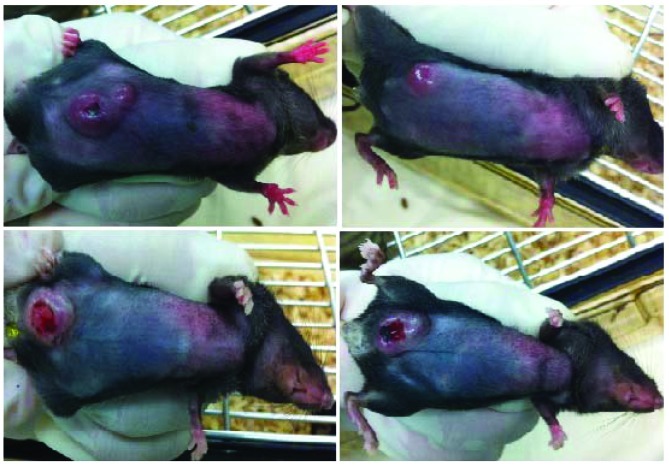
Development of tumors in female C57BL/6 mice after injection of oncogene plasmid DNA. C57BL/6 mice were coadministered HRAS, c-Myc or shp53 oncogene and transposase plasmids subcutaneously. In all the animals (12/12), nodular subcutaneous neoplasms were developed on average 30 days after injection. Aggressive growth was observed in each tumor.

Recipients (12/12) of the three transposons and the SB transposase developed single or coupled, nodular, subcutaneous neoplasms ~30 days after injection (Fig. 2). Each tumor grew rapidly from initial detection to eventually reach a large volume.

### Animal PET imaging

**Figure 3:**
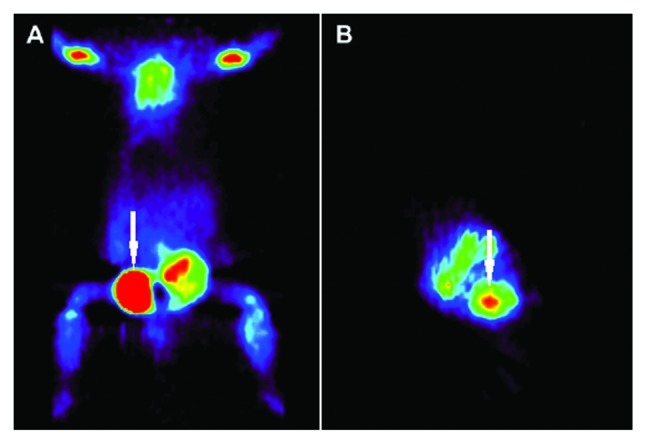
PET-CT scan of the tumor. PET-CT images of subcutaneous neoplasm. (A) Longitudinal CT section and (B) transverse CT section. The tumor is shown as a red-pinkish-colored small ball (arrows).

Different dimensions, both transverse and longitudinal, of PET-CT images for subcutaneous neoplasms were detected on the injection sites. The tumors are observed as a red-pinkish-colored small ball (Fig. 3).

### Gross and microscopic findings

**Figure 4:**
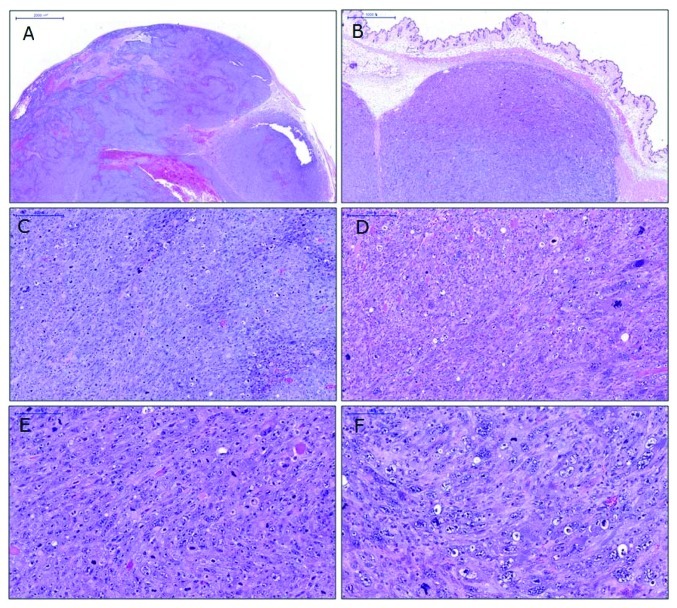
Microscopic images of a cut surface of each tumor in H&E staining. (A and B) Tumors had the same histological characteristic including clear demarcation, without metastasis (magnification, ×20). (C and D) Tumors generally consisted of highly pleomorphic and undifferentiated cells. Some tumors resemble epithelial cells, while others resemble mesenchymal cells. (E and F) The poorly differentiated cells were round to oval in shape, with a slightly basophilic cytoplasm, oval to elongated hyperchromatic nuclei and prominent nucleoli. Bizarre cells, with large and pale cytoplasm and a more prominent nuclei, were observed (magnification, ×200).

A well-demarcated, ovoid nodule was located in the subcutaneous soft tissue where the DNA plasmids were injected. Metastatic foci in non-injected sites were not observed. The tumor showed high cellularity with abundant mitoses and apoptosis. Both epithelial and mesenchymal components were evident. The tumor cells were undifferentiated with pleomorphic features characterized by round to oval cells with pale basophilic cytoplasm and hyperchromatic nuclei with prominent nucleoli (Fig. 4). Multinucleated giant cells were occasionally identified. Areas of necrosis were also evident in the central part of the tumor. However, the overlying skin and its associated adnexa, including hair follicles, sebaceous glands and mammary glands, did not exhibit dysplastic changes. All the tumors had the same morphological features.

### Immunohistochemical findings

**Figure 5:**
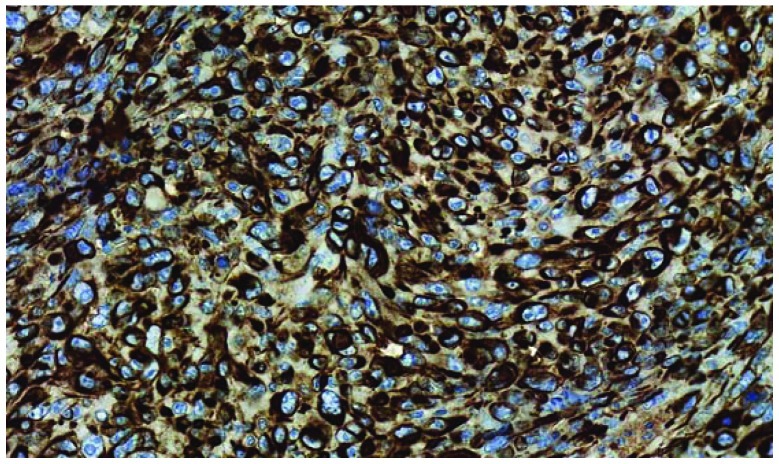
Pan-cytokeratin immunohistochemistry results of induced tumors. Tumors showing positive immunoreactivity to pan-cytokeratin (magnification, ×300).

As summarized in Table II, only pan-cytokeratin was found to be positively expressed by the tumor cells (Fig. 5), suggesting the epithelial origin of the tumor. The other markers were completely negative, except in the tumor-associated stroma. Consequently, we diagnosed this tumor as a sarcomatoid carcinoma.

### Identification of gene expression in the tumor

Expression of the c-Myc and HRAS oncogenes was demonstrated by immunohistochemical positivity for the c-Myc and HRAS antibodies, respectively. A positive signal was detected with an anti-GFP antibody, indicating expression of the shp53 DNA.

## Discussion

In the present study, we induced tumors in mice via the injection of transposons encoding three oncogenes and a plasmid-expressing transposase. The histological features of the induced tumor were poorly differentiated, thus we used immunohistochemical markers to characterize this lesion. Multinucleated giant cells were occasionally identified. Areas of necrosis were also identified in the central part of the tumor. Notably, the tumor expressed only pan-cytokeratin and all other markers used in this study were completely negative. As a result, we diagnosed the tumor as a sarcomatoid carcinoma.

To the best of our knowledge, there are no spontaneously occurring subcutis malignant epithelial tumors that morphologically resemble the tumor induced in this mouse study. The origin of this tumor remains unknown as the overlying epidermis and associated adnexa were not histologically dysplastic. Mammary tissues were also intact and normal mammary tissues were embedded within the tumor. This finding is consistent with the fact that mammary tissues lack response to the SB transposon system. Although this system has recently been shown to be able to induce various types of SB transposon-induced tumors, certain types of tumors such as those of the lung, mammary gland, prostate and pancreas, have not yet been generated with the SB transposon system in the mouse (10).

As the oncogenes were injected into the subcutis area, the origin of the tumor is assumed to be the skin adnexa, although this could not be precisely defined. c-Myc overexpression is associated with an undifferentiated phenotype in cultured astrocytes (11) and gastric carcinomas (12). In addition, c-Myc is downregulated during myogenic differentiation (13,14). HRAS overexpression is also associated with an anaplastic phenotype in mammary adenocarcinomas (15). Therefore, we hypothesize that injection of these oncogenes induced the growth of tumors with an undifferentiated phenotype.

The tumors induced in this study appeared to be of mesenchymal origin morphologically. Features of the tumors including undifferentiated pleomorphic cells characterized by hyperchromatic nuclei with prominent nucleoli and basophilic spindle-shaped cytoplasm resembled sarcomatous tumor. However, results of immunohistochemical staining showed this tumor to be carcinoma. p53 gene knockout is involved in presenting sarcomatoid features, as recent studies showed that tumor-suppressor gene mutations within the stroma allowed mesenchymal proliferation (16).

Insertional mutagenesis is commonly used in gene discovery studies in the field of oncology. This method is easy and more rapid compared to conventional genetic engineering (e.g., gene knockout). As a result, previous studies have focused on the use of the SB transposon system for insertional mutagenesis (17,18). However, detailed histological characterization of the induced tumors has not previously been performed. Thus, this study is, to the best of our knowledge, the first report of the histological and immunohistochemical identification of subcutaneous tumors induced by transposable elements.

## Figures and Tables

**Figure 1:**
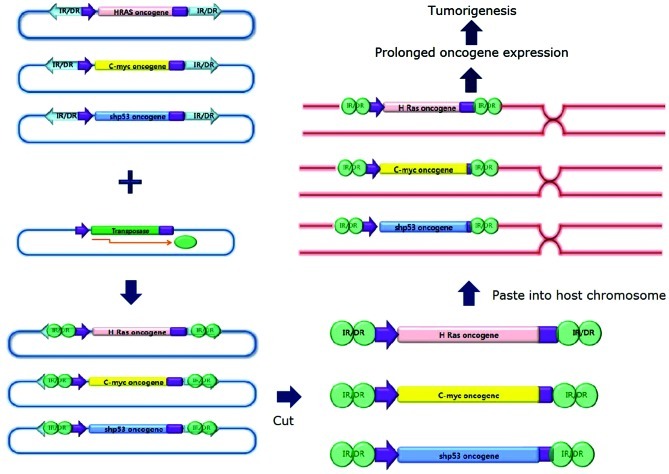
The concept of tumorigenesis using the Sleeping Beauty transposon system. The expressed transposase recognizes the specific sequence of the plasmids carrying an oncogene. It cuts and pastes each oncogene into the somatic chromosome, allowing for a longer expression of oncogenes in the host tissues. By using this system, tumors with a diverse combination of oncogenes can be readily induced easily.

**Table I:** The immunohistochemical panel.

Immunostains		Type	Manufacturer, location	Dilution
1	CD45	M	Abcam, Cambridge, UK	1:2,000
2	CD163	M	Abcam, Cambridge, UK	1:2,000
3	CD68	M	Abcam, Cambridge, UK	1:4,000
4	Desmin	M	Abcam, Cambridge, UK	1:1,000
5	Myogenin	M	Abcam, Cambridge, UK	1:100
6	Melanoma	M	Abcam, Cambridge, UK	1:1,000
7	S100	M	Abcam, Cambridge, UK	1:400
8	α-SMA	M	Abcam, Cambridge, UK	1:400
9	Pan-cytokeratin	M	Abcam, Cambridge, UK	1:3,000
10	Cytokeratin 7	P	Abcam, Cambridge, UK	1:4,000
11	Cytokeratin 20	M	Abcam, Cambridge, UK	1:2,000
12	MDM2	M	Abcam, Cambridge, UK	1:1,000
13	CDK4	M	Abcam, Cambridge, UK	1:1,000

M, monoclonal; P, polyclonal; SMA, smooth muscle actin.

**Table II:** Immunohistochemical profile.

Tissue of marker	IHC antibody	Reactivity
Epithelial tissue marker	Pan-cytokeratin	+
	Cytokeratin 7	−
	Cytokeratin 20	−
Muscular tissue marker	Myogenin	−
	Desmin	−
	α-SMA	−
Hematopoietic cell marker	CD45	−
	CD163	−
	CD68	−
Melnoma marker	Melanoma	−
	S100	−
Adipose tiusse marker	CDK4	−
	MDM2	−
Adenocarinoma marker	CEA	−

IHC, immunohistochemical; SMA, smooth muscle actin; CEA, carcinoembryonic antigen; +, positive; −, negative.
